# Prevalence and risk factors of hypotension associated with preload-dependence during intermittent hemodialysis in critically ill patients

**DOI:** 10.1186/s13054-016-1227-3

**Published:** 2016-02-23

**Authors:** Laurent Bitker, Frédérique Bayle, Hodane Yonis, Florent Gobert, Véronique Leray, Romain Taponnier, Sophie Debord, Alina Stoian-Cividjian, Claude Guérin, Jean-Christophe Richard

**Affiliations:** Service de Réanimation Médicale, Hôpital De La Croix Rousse, Hospices Civils de Lyon, 103 Grande Rue de la Croix Rousse, 69004 Lyon, France; Faculté de médecine Lyon-Est, Université de Lyon, Université Lyon I, 92 Rue Pasteur, 69007 Lyon, France; Institut Mondor de Recherche Biomédicale (IMRB), INSERM 955 Eq13, Faculté de Médecine de Créteil, 8, rue du Général Sarrail, 94010 Créteil, France; CREATIS, CNRS UMR 5220, INSERM 1044, INSA-Lyon, Université Lyon 1, 7 avenue Jean Capelle, 69621 Villeurbanne, France

**Keywords:** Acute kidney injury, Renal replacement therapy, Hemodynamics, Cardiac output, Circulatory failure, Cardiovascular monitoring

## Abstract

**Background:**

Hypotension is a frequent complication of intermittent hemodialysis (IHD) performed in intensive care units (ICUs). Passive leg raising (PLR) combined with continuous measurement of cardiac output is highly reliable to identify preload dependence, and may provide new insights into the mechanisms involved in IHD-related hypotension. The aim of this study was to assess prevalence and risk factors of preload dependence-related hypotension during IHD in the ICU.

**Methods:**

A single-center prospective observational study performed on ICU patients undergoing IHD for acute kidney injury and monitored with a PiCCO® device. Primary end points were the prevalence of hypotension (defined as a mean arterial pressure below 65 mm Hg) and hypotension associated with preload dependence. Preload dependence was assessed by the passive leg raising test, and considered present if the systolic ejection volume increased by at least 10 % during the test, as assessed continuously by the PiCCO® device.

**Results:**

Forty-seven patients totaling 107 IHD sessions were included. Hypotension was observed in 61 IHD sessions (57 %, CI_95%_: 47–66 %) and was independently associated with inotrope administration, higher SOFA score, lower time lag between ICU admission and IHD session, and lower MAP at IHD session onset. Hypotension associated with preload dependence was observed in 19 % (CI_95%_: 10–31 %) of sessions with hypotension, and was associated with mechanical ventilation, lower SAPS II, higher pulmonary vascular permeability index (PVPI) and dialysate sodium concentration at IHD session onset. ROC curve analysis identified PVPI and mechanical ventilation as the only variables with significant diagnostic performance to predict hypotension associated with preload dependence (respective AUC: 0.68 (CI_95%_: 0.53–0.83) and 0.69 (CI_95%_: 0.54–0.85). A PVPI ≥ 1.6 at IHD session onset predicted occurrence of hypotension associated with preload dependence during IHD with a sensitivity of 91 % (CI_95%_: 59–100 %), and a specificity of 53 % (CI_95%_: 42–63 %).

**Conclusions:**

The majority of hypotensive episodes occurring during intermittent hemodialysis are unrelated to preload dependence and should not necessarily lead to reduction of fluid removal by hemodialysis. However, high PVPI at IHD session onset and mechanical ventilation are risk factors of preload dependence-related hypotension, and should prompt reduction of planned fluid removal during the session, and/or an increase in session duration.

**Electronic supplementary material:**

The online version of this article (doi:10.1186/s13054-016-1227-3) contains supplementary material, which is available to authorized users.

## Background

Hypotension is a major complication of intermittent hemodialysis (IHD) in intensive care units (ICUs), and may impair efficiency of renal replacement therapy, increase mortality, and decrease renal recovery after acute kidney injury [1]. Although continuous renal replacement therapy techniques are now recommended for hemodynamically unstable patients, these may be replaced by IHD once hemodynamic stability has been achieved [1]. Even though dedicated ICU practice guidelines aiming to improve hemodynamic tolerance of IHD [2] are applied, hypotension rates ranging from 17 % to 56 % are still reported [2–6]. The common consequence of IHD-related hypotension is to discontinue the fluid removal for the rest of the IHD session, with the main consequence of impairing fluid balance control, which has been repeatedly shown as a major determinant of mortality in patients with septic shock or acute respiratory distress syndrome [7–9].

However, this management relies on the assumption that the underlying cause of hypotension is hypovolemia. Yet, several other determinants of hypotension during hemodialysis have been identified such as reduced cardiac output of various origins (hypocalcemia, diastolic dysfunction,…) or alterations of the vasomotor tone related to positive thermal balance, membrane/circuit bio-incompatibility or ionic imbalance, among others [10–13].

Previous studies have used the Swan-Ganz catheter to investigate hemodynamic effects of IHD [10, 12], but this technique is not reliable to predict preload dependence (cardiac output increase in response to fluid administration) during acute circulatory failure [14]. Several modern cardiac output monitoring devices with fast response time have now the potential to reliably classify hypotensive episodes as dependent or non-dependent on cardiac preload [15]. Passive leg raising (PLR) combined with continuous measurement of cardiac output is a highly reliable bedside method to identify preload dependence in a large variety of clinical settings in the ICU (spontaneous breathing and deeply sedated patients, regular or irregular cardiac rhythm) [16]. We hypothesize that this technique, by reliably classifying hypotension as related or unrelated to cardiac preload, would help the management of fluid removal during IHD. However, to date, no study has attempted to evaluate the prevalence of preload dependence-related hypotension during IHD in ICU patients.

The aim of this study was to assess the prevalence of hypotension and preload dependence-related hypotension episodes in critically ill patients undergoing IHD. Secondary objectives were (1) to identify risk factors for hypotension and hypotension related to preload dependence during IHD in ICU and (2) to assess diagnostic performance of variables associated with hypotension related to preload dependence.

## Methods

### Study design

This is a prospective observational single-center study performed between May 1, 2012 and May 31, 2014 in a 15-bed medical ICU. The study was approved by an ethics committee (CECIC Rhône-Alpes-Auvergne, Grenoble, France, IRB 5921), which waived the requirement for informed consent given the observational nature of the study.

### Patients

To be eligible the subjects had to fulfill all the following inclusion criteria: age of 18 years or older, staying in our ICU, PiCCO® device (Pulsion Medical Systems, Feldkirchen, Germany) already in place for acute circulatory failure, and acute kidney injury requiring renal replacement therapy with IHD [1]. Exclusion criteria were pregnancy, lower limb amputation, excessive nurse’s workload, known obstruction of inferior vena cava, and former inclusion in the study during a previous ICU admission. Multiple IHD sessions per patient during the same ICU stay were possibly eligible, should inclusion and exclusion criteria be fulfilled at IHD session onset.

### IHD sessions

In our ICU the IHD sessions are managed by a specific team of technical nurses, the indication and settings of IHD sessions being the responsibility of the clinician in charge of the patient, in accordance with current practice guidelines [1] (continuous renal replacement therapy being used as a first-line technique in hemodynamically instable patients). IHD was performed with either INNOVA (Gambro Hospal, Meyzieu, France) or AK200 Ultra S (Gambro AB, Lund, Sweden) hemodialysis generators, Nephral ST 300 AN69ST dialysate membranes (Gambro Hospal, Meyzieu, France), and dialysate concentrate solutions with 1.75 mmol/L calcium concentration. IHD settings regarding blood and dialysate flow rate, dialysate temperature and dialysate sodium concentration were prescribed by the physician in charge, according to published practice guidelines [2].

### Hemodynamic management

Arterial and central venous blood pressures were continuously monitored, by using arterial femoral and jugular vein catheters, respectively, connected to Intellivue MP40 monitor equipped with the PiCCO® technology module (Philips Healthcare, Andover, MA, USA). Cardiac output was assessed using the PiCCO® device, calibrated with the transpulmonary thermodilution technique, using a triplicate intravenous infusion of 15 mL cold serum saline, immediately before the IHD session. Cardiac output was then continuously monitored using pulse contour analysis with the PiCCO® device during the sessions, using the pre-IHD thermodilution cardiac output value for calibration.

Mean arterial pressure (MAP) was targeted between 65 and 75 mm Hg in patients under vasopressor therapy. Hypotension was defined as the first occurrence of MAP below 65 mm Hg during the session. Once hypotension occurred, a PLR test was performed from the supine position by lifting the lower limbs at 45° for 1 minute. The test was also performed before the initiation of the IHD session. Preload dependence was deemed present if stroke volume increased by at least 10 % during the PLR. The physician response to preload-dependent hypotension, regarding fluid administration, reassessment of water balance or IHD session duration, was not protocolized.

### Data collection

The following variables were recorded at inclusion: demographic and anthropometric data, time of ICU admission, admission category, Simplified Acute Physiology Score (SAPS) II [17] and reason for PiCCO® monitoring.

The following variables were recorded at IHD session onset: Sequential Organ Failure Assessment (SOFA) score [18], heart rate, MAP, central venous pressure (CVP), cardiac index, extravascular lung water index, pulmonary vascular permeability index (PVPI), vasopressor administration and dose, inotrope administration, arterial lactate, need for mechanical ventilation and IHD settings.

### End points

Primary end points were the prevalence of hypotension and hypotension associated with preload dependence as assessed by passive leg raising, respectively.

Secondary end points were the identification of risk factors for both hypotension and hypotension related to preload dependence, and the diagnostic performance of variables associated with hypotension related to preload dependence during IHD.

### Statistical analysis

Statistical analyses were performed using R software [19]. A *p* value below 0.05 was chosen for statistical significance. The statistical unit was the IHD session. Power of the study was computed using the normal approximation confidence interval method [20]. We calculated that with a sample size of at least 96 IHD sessions, the study would provide at worst a ±10 % precision in the 95 % confidence interval of the prevalence of hypotension and hypotension related to preload dependence during IHD. Medians and interquartile ranges were reported for continuous variables, and counts in each category with corresponding percentages are given for categorical variables. Ninety-five percent confidence intervals (CI_95%_) for proportions were calculated using the Wilson score test.

Since some patients were studied during several IHD sessions, quantitative variables were compared between groups of sessions (with hypotension vs. without hypotension, and with hypotension and preload dependence vs. with hypotension without preload dependence) with a linear mixed model, using group as variable with a fixed effect, and patient as variable with a random effect [21]. Qualitative variables were compared similarly with a mixed logistic regression model, using patient as variable with a random effect.

Quantitative and qualitative variables associated with hypotension with a *p* value below 0.1 in univariate analysis (using a mixed logistic regression model with patient as variable with a random effect) were selected for inclusion in a multivariable mixed logistic regression model, using backward stepwise descending selection [22]. Low prevalence of hypotension with preload dependence precluded the use of a multivariate logistic regression model.

Diagnostic performance of variables associated with preload-dependent hypotension was tested by computation of the area under the curve (AUC) of the receiver operating characteristic (ROC) curve [23]. The optimal cutoff values were computed using the Youden J statistic method [24].

## Results

### Population

During the study inclusion period, 1462 patients were admitted to the ICU, and 47 (median age 69 [63–78] years) were included in the study (see Additional file 1). Median SAPS II score was 53 [39–61], 32 patients were male (68 %), and 45 patients (96 %) were admitted with a medical admission category. Justification for PiCCO® monitoring was septic shock in 26 patients (55 %), cardiogenic shock in 10 patients (21 %), hemorrhagic shock in 4 patients (9 %), non-septic vasoplegic shock in 4 patients (9 %) and other shocks in 3 patients (6 %). Patients’ characteristics at the onset of IHD session are reported in Table 1.

**Table 1:** Patients’ characteristics at hemodialysis session onset

Characteristics	Overall population
Mechanical ventilation	39 (36 %)
SOFA score	8 [7–11]
Vasopressor administration	53 (50 %)
Vasopressor dose (μg.kg^-1^.min^-1^)	0.00 [0.00–0.15]
Inotropes administration	9 (8 %)
Arterial lactate (mmol.L^-1^)	1.4 [1.1–1.8]
Elevated lactates above upper laboratory limit (2.2 mmol.L^-1^)	14 (13 %)
Heart rate (min^-1^)	90 [80–98]
Mean arterial pressure (mm Hg)	75 [68–80]
Mean arterial pressure below 65 mm Hg	8 (7 %)
Central venous pressure (mm Hg)	8 [6–12]
Cardiac index (L.min^-1^.m^-2^)	3.5 [2.9–4.0]
Indexed systemic vascular resistance (dyne.s.cm^-5^)	1525 [1324–1864]
Extravascular lung water (mL.kg^-1^ PBW)	7.9 [6.0–9.9]
Extravascular lung water > 7 mL.kg^-1^ PBW	64 (60 %)
Pulmonary vascular permeability index	1.6 [1.3–2.0]

Data are median [first quartile – third quartile] or number of sessions (percentage of the total number of sessions studied)

*PBW* predicted body weight, *SOFA* Sequential Organ Failure Assessment score

### IHD sessions

One hundred and seven IHD sessions were studied. Median number of IHD sessions per patient was three [2–5]. Twenty-seven patients underwent two or more IHD sessions. Details regarding IHD sessions are reported in Table 2. Missing values for each variable are reported in Additional file 2. Compliance to practice guidelines [2] to prevent hemodynamic instability during IHD sessions was high (see Additional file 3).

**Table 2:** Characteristics of intermittent hemodialysis sessions

Characteristics	Overall population
Number of IHD sessions	107
Number of IHD sessions per patient	3 [2–5]
Time between ICU admission and first IHD session (days)	18 [10–30]
Duration of IHD sessions (min)	240 [240–245]
Total fluid removal per session (mL)	2620 [1990–3240]
Fluid removal per hour (mL.H^-1^)	655 [453–824]
Dialyzer blood flow rate at IHD session onset (mL.min^-1^)	250 [250–272]
Dialysate flow rate at IHD session onset (mL.min^-1^)	500 [500–625]
Dialysate temperature at IHD session onset (°C)	36 [36–36]
Dialysate sodium concentration at IHD session onset (mmol.L^-1^)	145 [144–150]

Data are medians [first quartile – third quartile]

*IHD* intermittent hemodialysis

### Hypotension

**Fig. 1:**
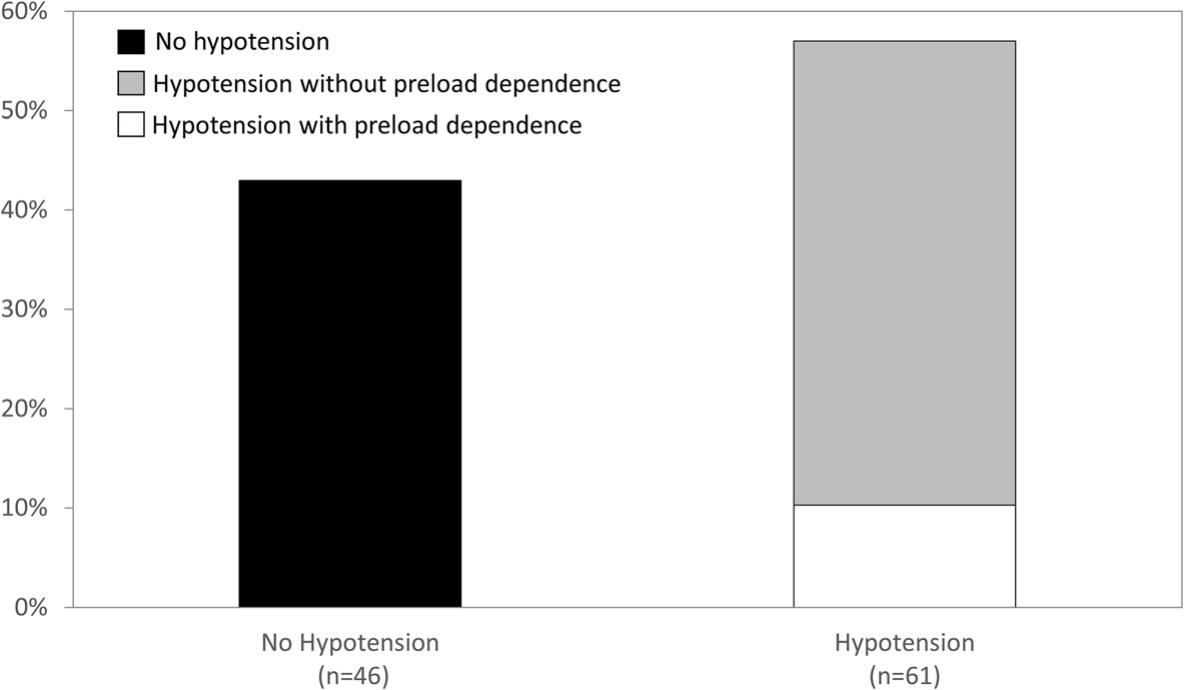
Prevalence of hypotension during intermittent hemodialysis sessions

Hypotension was observed during 61 (57 %, CI_95%_: 47–66 %) IHD sessions (Fig. 1), with a median time from IHD session onset of 35 [15–95] minutes. Effective fluid removal at hypotension onset was 300 [30–950] mL. Comparisons between IHD sessions with hypotension and without hypotension are presented in Table 3.

**Table 3:** Comparisons between hemodialysis sessions with and without hypotension

Characteristics	No hypotension during session	Hypotension during session
	(n = 46)	(n = 61)
Age	70 [64–79]	67 [58–78]
Male gender	38 (83 %)	47 (77 %)
SAPS II	53 [40–62]	53 [37–60]
Reasons for PiCCO® monitoring		
• Septic shock	30 (65 %)	34 (56 %)
• Cardiogenic shock	7 (15 %)	10 (16 %)
• Other	9 (20 %)	17 (28 %)
Time between ICU admission and IHD session (day)	20 [12–34]	16 [9–29]
SOFA score at the day of IHD session	7 [6–9]	10 [8–11]^†^
Mechanical ventilation at IHD session onset	18 (39 %)	21 (34 %)
Inotrope at IHD session onset	7 (15 %)	2 (3 %)
Vasopressor at IHD session onset	20 (43 %)	33 (54 %)
Vasopressor dose at IHD session onset (μg.kg^-1^.min^-1^)	0 [0–0.10]	0.04 [0–0.19]
Heart rate at IHD session onset (min^-1^)	89 [80–98]	90 [81–98]
MAP at IHD session onset (mm Hg)	78 [71–83]	72 [67–79]^†^
CVP at IHD session onset (mm Hg)	9 [7–13]	8 [5–11]
CI at IHD session onset (L.min^-1^.m^-2^)	3.7 [3.0–3.9]	3.4 [2.8–4.0]
ISVR at IHD session onset (dyne.s.cm^-5^)	1561 [1366–1907]	1501 [1262–1764]
EVLWI at IHD session onset (mL.kg^-1^ PBW)	8.2 [6.1–10.9]	7.7 [6.0–9.7]
PVPI at IHD session onset	1.5 [1.2–2.0]	1.6 [1.4–2.0]
Preload dependence at IHD session onset	1 (2 %)	4 (7 %)
Arterial lactates (mmol.L^-1^)	1.4 [1.1–1.8]	1.4 [1.1–1.8]
Elevated lactates above upper laboratory limit (2.2 mmol.L^-1^)	4 (9 %)	10 (16 %)
Duration of IHD sessions (min)	240 [240–240]	240 [240–250]
Total fluid removal during IHD session (mL)	3000 [2162–3370]	2400 [1670–3200]
Fluid removal per hour during IHD session (mL.H^-1^)	756 [541–820]	575 [400–829]
Dialyzer blood flow rate at IHD session onset (L.min^-1^)	250 [250–270]	250 [250–275]
Dialysate flow at IHD session onset (mL.min^-1^)	500 [500–600]	500 [500–700]
Dialysate temperature at IHD session onset (°C)	36 [36–36]	36 [36–36]
Dialysate sodium concentration at IHD session onset (mmol.L^-1^)	145 [141–148]	145 [145–150]

Data are medians [first quartile – third quartile] or number (percentage)

*CI* cardiac index, *CVP* central venous pressure, *MAP* mean arterial pressure, *EVLWI* extravascular lung water, *IHD* intermittent hemodialysis, *ISVR* indexed systemic vascular resistance, *PBW* predicted body weight, *PVPI* pulmonary vascular permeability index, *SAPS II* Simplified Acute Physiology Score II, *SOFA* Sequential Organ Failure Assessment score

^†^ *p* < 0.05 between groups

In sessions complicated by at least one hypotensive episode, SOFA score was significantly higher (10 [8–11] vs. 7 [6–9], *p* = 0.008), and mean arterial pressure at IHD session onset was significantly lower (72 mm Hg [67–79] vs. 78 mm Hg [71–83], *p* < 0.01), as compared to sessions without hypotensive episode. The prevalence of preload dependence at IHD onset did not differ between hypotensive and non-hypotensive sessions (Table 3). Vasopressor dose, other hemodynamic parameters and IHD settings were not statistically different between hypotensive and non-hypotensive sessions (Table 3).

We performed a multivariate analysis of variables associated with hypotension during IHD sessions, using the following variables (see Additional file 4): time between ICU admission and IHD session, SOFA score, inotrope administration at IHD session onset, MAP at IHD session onset, and sodium conductivity at IHD session onset. SOFA score, time between ICU admission and IHD session, MAP and inotrope administration at IHD session onset were significantly and independently associated with hypotension status during IHD sessions (Table 4).

**Table 4:** Risk factors associated with at least one hypotensive episode retained in the multivariable model

Risk factor	Odds ratio	CI_95%_ of odds ratio	*p* value
SOFA score, per one point increment	1.34	1.13–1.76	<0.01
MAP at IHD session onset, per one mm Hg increment	0.93	0.84–0.98	0.03
Inotrope at IHD session onset			0.04
• No^†^	1		
• Yes	0.13	0.00–0.76	
Time between ICU admission and IHD session (day), per one point increment	0.96	0.91–0.99	0.04

CI_95%_ 95 % confidence interval, *IHD* intermittent hemodialysis *ICU* intensive care unit, *MAP* mean arterial pressure, *SOFA* Sequential Organ Failure Assessment score

^†^No inotrope at IHD onset was the reference

### Hypotension with preload dependence

Two sessions were not assessed for preload dependence during hypotension and were therefore excluded from subsequent analysis. Hypotension associated with preload dependence was observed in 11 IHD sessions (19 %, CI_95%_: 10–31 % of sessions with hypotension). Comparisons in patients’ characteristics and IHD settings associated with preload- and non-preload-dependent hypotension are available in Table 5.

**Table 5:** Characteristics of hypotensive episodes with and without preload dependence

Characteristics	Hypotension without preload dependence	Hypotension with preload dependence
	(n = 48)	(n = 11)
Age	70 [58–78]	64 [59–69]
Male gender	38 (79 %)	7 (64 %)
SAPS II	54 [37–60]	49 [41–55]^†^
Reasons for PiCCO® monitoring		
• Septic shock	25 (52 %)	8 (73 %)
• Cardiogenic shock	8 (17 %)	2 (18 %)
• Other	15 (31 %)	1 (9 %)
Time between ICU admission and IHD session (day)	18 [10–30]	9 [6–15]
SOFA score at the day of IHD session	9 [7–11]	10 [9–10]
Mechanical ventilation at IHD session onset	13 (27 %)	8 (73 %)^†^
Inotrope at IHD session onset	1 (2 %)	1 (9 %)
Vasopressor at IHD session onset	25 (52 %)	7 (64 %)
Vasopressor dose at IHD session onset (μg.kg^-1^.min^-1^)	0.04 [0–0.19]	0.01 [0–0.13]
Heart rate at IHD session onset (min^-1^)	88 [79–96]	92 [83–108]^†^
MAP at IHD session onset (mm Hg)	70 [66–77]	72 [72–80]
CVP at IHD session onset (mm Hg)	8 [5–11]	8 [6–10]
CI at IHD session onset (L.min^-1^.m^-2^)	3.4 [2.8–3.9]	3.6 [3.2–4.1]
ISVR at IHD session onset (dyne.s.cm^-5^)	1497 [1294–1711]	1440 [1160–1636]
EVLWI at IHD session onset (mL.kg^-1^ PBW)	8.8 [6.5–12.7]	10.9 [9.0–12.4]
PVPI at IHD session onset	1.5 [1.3–1.9]	2.0 [1.6–2.0]^†^
Preload dependence at IHD session onset	3 (6 %)	1 (9 %)
Arterial lactates (mmol.L^-1^)	1.3 [1.1–1.6]	1.5 [1.4–1.9]
Elevated lactates above upper laboratory limit (2.2 mmol.L^-1^)	8 (17 %)	1 (9 %)
Duration of IHD sessions (min)	240 [240–250]	228 [228–240]
Total fluid removal during IHD session (mL)	2580 [1738–3400]	2250[390–2990]
Fluid removal during IHD session (mL.H^-1^)	576 [411–748]	562 [95–748]
Dialyzer blood flow rate at IHD session onset (L.min^-1^)	250 [250–271]	250 [250–295]
Dialysate flow at IHD session onset (mL.min^-1^)	500 [500–700]	700 [500–700]
Dialysate temperature at IHD session onset (°C)	36 [36–36]	36 [36–36]
Dialysate sodium concentration at IHD session onset (mmol.L^-1^)	145 [144–149]	150 [145–150]^†^
Time between IHD session onset and hypotension (min)	30 [14–94]	60 [25–135]
Cumulative fluid removal at time of hypotension (mL)	265 [10–825]	300 [70–1195]

Data are medians [first quartile – third quartile] or number (percentage)

*CI* cardiac index, *CVP* central venous pressure, *EVLWI* extravascular lung water, *IHD* intermittent hemodialysis, *ISVR* indexed systemic vascular resistance, *MAP* mean arterial pressure, *PBW* predicted body weight, *PVPI* pulmonary vascular permeability index, *SAPS II* Simplified Acute Physiology Score II, *SOFA* Sequential Organ Failure Assessment score

^†^ *p* < 0.05 between groups

In the preload-dependent hypotensive group, SAPS II score was significantly lower, while heart rate, PVPI, dialysate sodium concentration at IHD session onset were significantly higher, and mechanical ventilation was significantly more frequent (73 % vs. 27 %, *p* = 0.01). Among these variables, ROC curve analysis identified PVPI at IHD onset and mechanical ventilation as the only variables with significant diagnostic performance, as assessed by their AUC significantly different from 0.5 (Table 6). A PVPI ≥ 1.6 at IHD session onset predicted occurrence of hypotension associated with preload dependence during IHD with a sensitivity of 91 % (CI_95%_: 59–100 %), and a specificity of 53 % (CI_95%_: 42–63 %).

**Table 6:** Diagnostic performance of parameters associated with preload dependence-related hypotension in univariate analysis

Parameter	AUC [CI_95%_]	Optimal cutoff	Se [CI_95%]_	Sp [CI_95%_]	FP	FN	PPV	NPV	PLR	NLR	Youden index
PVPI at IHD onset	0.68^†^ [0.53–0.83]	1.6	0.91 [0.59–1.00]	0.53 [0.42–0.63]	42	1	0.19	0.98	1.93	0.17	0.44
Mechanical ventilation at IHD onset	0.69^†^ [0.54–0.85]	NA	0.64 [0.32–0.88]	0.75 [0.65–0.83]	24	4	0.23	0.95	2.55	0.15	0.39
HR at IHD onset (min^-1^)	0.60 [0.38–0.81]	105	0.36 [0.11–0.69]	0.91 [0.83–0.96]	9	7	0.31	0.93	3.88	0.70	0.27
Dialysate sodium concentration at IHD onset (mmol.L^-1^)	0.63 [0.45–0.81]	145	0.82 [0.48–0.98]	0.45 [0.45–0.55]	53	2	0.15	0.96	1.48	0.41	0.27
SAPS II	0.46 [0.30–0.62]	38	0.91 [0.59–1.00]	0.27 [0.19–0.37]	53	2	0.13	0.96	1.24	0.34	0.18

*AUC* area under the curve, *CI*_*95%*_ 95 % confidence interval, *FP* false positive, *FN* false negative, *HR* heart rate, *NA* not applicable, *NLR* negative likelihood ratio*, NPV* negative predictive value, *PLR* positive likelihood ratio, *PPV* positive predictive value, *PVPI* pulmonary vascular permeability index, *SAPS II* Simplified Acute Physiology Score II, *Se* sensitivity, *Sp* specificity

^†^Area under the curve significantly different from 0.5

**Fig. 2:**
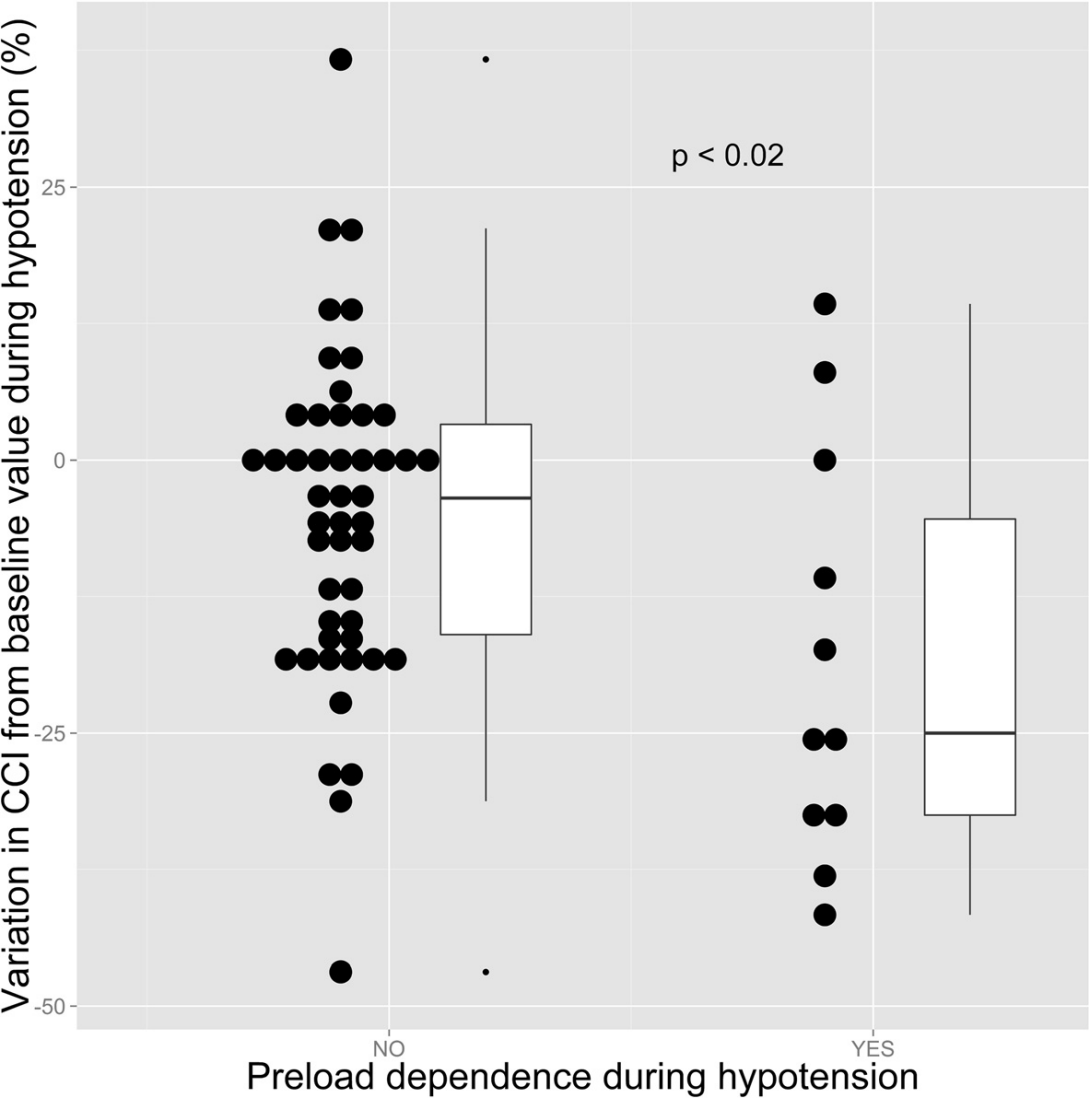
Variation of CCI from baseline value during hypotension as a function of preload dependence status. *Black circles* are individual values. *CCI* continuous cardiac index

Both groups also differed at hypotension onset regarding continuous cardiac index variation from IHD onset, with a significantly greater decrease in the preload-dependent group (−25 % [−33 % to −5 %] vs. −3 % [−16 % to 3 %], *p* < 0.02, Fig. 2).

## Discussion

This study is the first to assess the prevalence of preload dependence during IHD in the ICU. The main findings are that (1) the majority of first hypotension episodes occurring during IHD are not related to preload dependence and hence to fluid removal by IHD; (2) hypotension during IHD is unrelated to dialysis settings when ICU dedicated practice guidelines to prevent hemodynamic instability are applied, and is mainly related to preexistent cardiovascular and organ dysfunction; (3) high PVPI and mechanical ventilation are risk factors for preload dependence-related hypotension during IHD and may prompt, if identified at IHD onset, reduction of planned fluid removal during the session, or increased session duration.

### Statistical methodology

The use of IHD session as a statistical unit (and not patient) was purposely chosen, in accordance with the study aim (prevalence of hypotension during IHD sessions), and in line with previous studies addressing hypotension during IHD [3, 5, 6]. As a consequence, it was possible to obtain multiple measurements per patient, leading to the choice of mixed effects statistical modeling, which allows accurate analysis of unbalanced repeated measures data [25], since correlation between repeated observations is taken into account by the model.

### Hypotension during IHD sessions

Hypotensive episodes were present in 57 % of IHD sessions in the present study, a rate in the upper range of previous ICU studies, having documented hypotension rates ranging from 17 to 56 % [2–6]. As compared to those studies, the present study is characterized by a higher prevalence of patients requiring vasopressors, similar SAPS II scores, and a lower percentage of patients requiring mechanical ventilation (see Additional file 5). While dissimilarities in case mix or dialysis settings between studies may explain this difference, heterogeneity between studies regarding the criteria defining hypotension may also be an alternative explanation (see Additional file 5), since there is no universally accepted standard to define hemodynamic instability during IHD in the ICU. However, despite differences in study settings, the present study identified similar independent risk factors for hypotension during IHD (e.g., lower arterial blood pressure at IHD onset and SOFA score [3]), confirming that patient-related factors of organ dysfunction are strongly associated with cardiovascular tolerance of IHD sessions. The association between time between ICU admission and IHD session and hypotension occurrence (higher time lag being identified as associated with less hypotension by an odds ratio significantly lower than 1) may be viewed as an incentive to favor continuous renal replacement therapy techniques at the initial phase of ICU stay, in line with current recommendations suggesting the use of this technique for hemodynamically unstable patients [1].

The protective role of cardiac inotrope regarding hypotension occurrence is unclear, and could be related to a specific subset of cardiac patients (although non-identified in our population analysis), or a direct effect of this drug preventing low cardiac output. Conversely and in accordance with a recent study [3], we were unable to identify any association between hypotension occurrence during IHD and IHD settings. This may be a consequence of a strong adherence to IHD practice guidelines aiming to improve IHD hemodynamic tolerance [2] in both studies.

### Association between hypotension and preload dependence during IHD sessions

The most striking result of this study is that a very limited proportion of hypotensive patients had a positive response to the PLR test and hence were deemed preload dependent (19 % of first hypotensive episodes), suggesting that the vast majority of first hypotensive episodes during IHD are not related to hypovolemia. We were unable to compare this finding with others, due to the lack of previously published studies on that matter. Our data suggests that the main cause of hypotension during IHD in our study is an alteration in the vasomotor tone, since median change from pre-IHD value in continuous cardiac index was close to zero during hypotension in the non-preload-dependent hypotension group. Conversely and accordingly with the Franck-Starling mechanism, median change in continuous cardiac index between pre-IHD and hypotension significantly shifted toward more negative values in the preload-dependent group as compared to the non-preload-dependent group (Fig. 2).

Among the five clinical variables associated with preload dependence-related hypotension, our analysis suggests that only PVPI at IHD onset and mechanical ventilation have useful diagnostic performance to identify at-risk patients at IHD onset, although the lack of multivariate analysis precludes any strong inference regarding these variables. High PVPI and a larger proportion of patients under mechanical ventilation may be related to increased risk of preload dependence hypotension by (1) respectively promoting increased plasma capillary leak in the pulmonary vasculature and (2) cardiopulmonary interaction by alteration of systemic venous return. Increased heart rate at IHD onset in the preload-dependent hypotension group could be related to preexistent hypovolemia, although the lack of significant difference in the proportion of preload-dependent patients at IHD onset strongly argues against this hypothesis. The relationship between increased dialysate sodium concentration and preload dependence-related hypotension is unclear, as this should promote interstitium-to-plasma water transfer and increase IHD hemodynamic tolerance [2]. The association between lower SAPS II and occurrence of preload dependence-related hypotension is also unclear.

### Limitations

Some limitations of the present study should be acknowledged. First, the single-center nature of this study could limit its generalizability, as patient case mix, selection of a subgroup of patients monitored by the PiCCO® device, local practice of using IHD as a second-line modality following continuous renal replacement therapy techniques after improvement of hemodynamic status [1], or main end point restricted to the first episode of hypotension, are center or study specific. Nevertheless, the strong adherence to practice guidelines aiming to limit hemodynamic instability during IHD sessions [2] ensures that IHD management of study patients reflects general practice, although a selection bias cannot be ruled out.

Second, the lack of significant difference regarding effective fluid removal by IHD between hypotensive episodes related or unrelated to preload dependence could have been related to a reevaluation of planned fluid removal by the physician in charge during IHD, in relation with hemodynamic instability. However, similar results were obtained using planned fluid removal (data not shown).

Third, PLR was performed from the supine position, while one study suggested that starting from the semi-recumbent position increases the diagnosis performance of the PLR test [26], since it may mobilize venous blood from the splanchnic compartment in addition to venous blood from the lower limbs. However, this study [26] was restricted to volume-responder patients, and respective sensitivity and specificity of both tests could not be computed. To the contrary, a meta-analysis of 23 studies did not find any effect of the starting position on diagnosis accuracy of the PLR test [27]. However, assuming a conservative assumption of a lower sensitivity of the PLR test performed from the supine position (although not identified by meta-analysis), we cannot rule out that the prevalence of preload dependence was underestimated in the present study.

Four, due to the non-interventional nature of the study, we are unable to conclude regarding the effect of therapeutic interventions after hypotension onset (e.g., of change in fluid removal settings, fluid administration, vasopressor change…), and their effect on the PLR test result.

Five, cardiac index was not assessed by the thermodilution technique during hypotension, but solely by pulse contour analysis, whose reliability may be altered by acute changes in the vasomotor tone. However, it was previously shown in 32 patients [28] that the accuracy of continuous cardiac output assessed by the PiCCO® device remained clinically acceptable within 1 hour of the calibration procedure (percentage error lower than 30 %) despite significant change in the vascular tone (as defined by a greater than 15 % variation in systemic vascular resistance). Therefore, the short delay between PiCCO® calibration and hypotension occurrence (35 [15–95] min) in the present study and significant decrease in cardiac index during preload-dependent hypotension as predicted by the Franck-Starling mechanism, favors the reliability of the measurement in most patients.

Six, the relatively low number of preload dependence-associated hypotension episodes in this study weakens the relevance of associated variables as it prevented multivariate analysis of variables associated with preload-dependent hypotension.

### Clinical implications

The low percentage of preload dependence-related hypotension, should it be confirmed in a wider ICU setting, suggests that hypotension occurring during IHD sessions should not systematically lead to discontinuation of fluid removal, but prompt hemodynamic evaluation of cardiovascular status. This should be achieved by using a hemodynamic device which can reliably classify patients regarding their preload dependence status (e.g., echocardiography, esophageal Doppler, or other cardiac output monitors with the ability to provide real-time measurement of cardiac output during a PLR maneuver [29]…). Furthermore, the high sensitivity of the PVPI at IHD onset to predict occurrence of preload dependence-related hypotension may help to identify at-risk patients and may prompt downward revision of the total planned fluid removal during the IHD session, or an increase in IHD session duration. This should obviously be confirmed in future studies, before implementation in the clinical setting.

## Conclusions

In the context of our center, the majority of hypotensive episodes occurring during intermittent hemodialysis are unrelated to preload dependence and should not necessarily lead to reduction of fluid removal by hemodialysis, but should prompt hemodynamic evaluation of cardiovascular status to reliably classify patients regarding their preload dependence status. Lower arterial pressure at dialysis onset, higher SOFA score, and shorter time lag between ICU admission and IHD session are the main risk factors for hypotension during IHD, while high PVPI at dialysis onset and mechanical ventilation are risk factors for preload dependence-related hypotension during IHD and may prompt, if identified at IHD onset, reduction of planned fluid removal during the session, or an increase in session duration.

## Key messages

- Lower arterial pressure at dialysis onset, higher SOFA score, and shorter time lag between ICU admission and IHD session are the main risk factors for hypotension during intermittent hemodialysis
- The majority of hypotensive episodes occurring during intermittent hemodialysis are unrelated to preload dependence and should not necessarily lead to reduction of fluid removal by hemodialysis.
- Identified risk factors for preload dependence-associated hypotension during IHD are high pulmonary vascular permeability index at hemodialysis onset and mechanical ventilation, and may prompt reduction of planned fluid removal during the session, or an increase in session duration.

## Additional files

Additional file 1:
**Study flow chart.** Description of data: study flow chart. (PDF 145 kb) Additional file 2:
**Missing values for each studied variable.** Description of data: proportions are computed with either total number of patients (n = 47), total number of hypotensive episodes (n = 61) or total number of IHD sessions (n = 107), as needed. (PDF 111 kb) Additional file 3:
**Compliance to practice guidelines of intermittent hemodialysis in intensive care units.** Description of data: compliance to practice guidelines of intermittent hemodialysis in intensive care units according to reference [2]. Data are number of sessions (percentage of the total number of sessions studied). (PDF 422 kb) Additional file 4:
**Univariate analysis of risk factors associated with at least one hypotensive episode.** Description of data: univariate analysis of risk factors associated with at least one hypotensive episode. (PDF 305 kb) Additional file 5:
**Hypotension rates during intermittent hemodialysis in intensive care units in published studies.** Description of data: hypotension rates during intermittent hemodialysis in intensive care units in published studies. (PDF 113 kb)

## Abbreviations

AUC: Area under the curve
CI_95%_: 95 % Confidence interval
CVP: Central venous pressure
ICU: Intensive care unit
IHD: Intermittent hemodialysis
MAP: Mean arterial pressure
PLR: Passive leg raising
PVPI: Pulmonary vascular permeability index
ROC: Receiver operating characteristic
SAPS II: Simplified Acute Physiology Score II
SOFA: Sequential Organ Failure Assessment
