# Supplementary material for: Prevalence and risk factors of hypotension associated with preload-dependence during intermittent hemodialysis in critically ill patients
Source: Crit Care. 2016 Feb 23;20:44. doi: 10.1186/s13054-016-1227-3 (PMC4765055; doi:10.1186/s13054-016-1227-3)
Supplement: Additional file 1: — Study flow chart. Description of data: study flow chart. (PDF 145 kb) [file 13054_2016_1227_MOESM1_ESM.pdf]

File name: Additional file 1

File format: .pdf

Title: Study flow chart.

Description of data: Study flow chart.

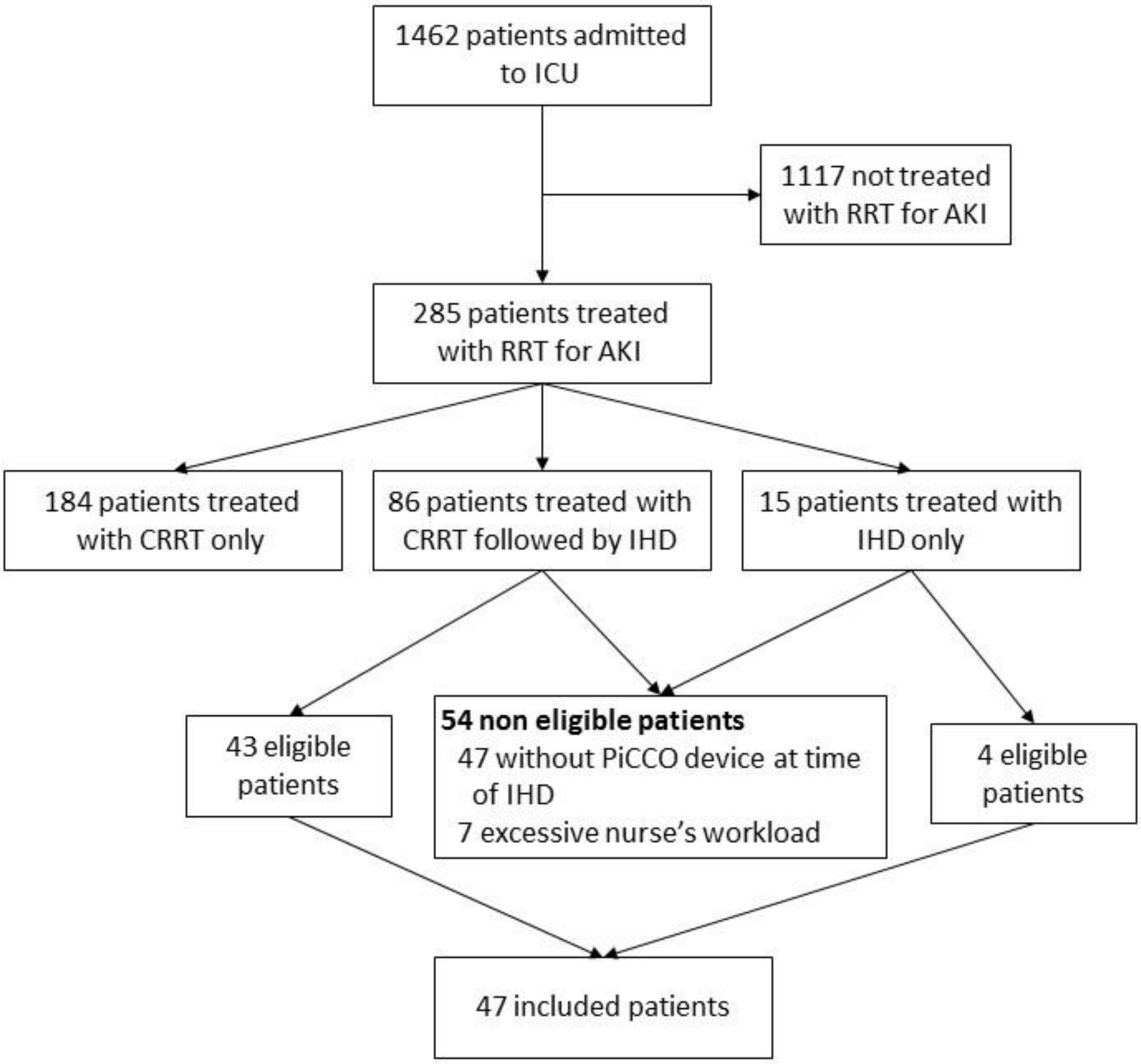

AKI = acute kidney injury; CRRT = continuous renal replacement therapy; ICU = intensive care unit; IHD = intermittent hemodialysis; RRT = renal replacement therapy.
