# Supplementary material for: Prevalence and risk factors of hypotension associated with preload-dependence during intermittent hemodialysis in critically ill patients
Source: Crit Care. 2016 Feb 23;20:44. doi: 10.1186/s13054-016-1227-3 (PMC4765055; doi:10.1186/s13054-016-1227-3)
Supplement: Additional file 2: — Missing values for each studied variable. Description of data: proportions are computed with either total number of patients (n = 47), total number of hypotensive episodes (n = 61) or total number of IHD sessions (n = 107), as needed. (PDF 111 kb) [file 13054_2016_1227_MOESM2_ESM.pdf]

File name: Additional file 2

File format: .pdf

Title: Missing values for each studied variable.

Description of data: Proportions are computed with either total number of patients (n=47), total number of hypotensive episodes (n=61) or total number of IHD sessions (n=107), as needed.

Variable	n	Proportion
Age	0	0%
Gender	0	0%
SAPSII	0	0%
Reason for PiCCO monitoring	0	0%
Mechanical ventilation	0	0%
Inotrope administration	0	0%
SOFA score	0	0%
Vasopressor administration	0	0%
Vasopressor dose at IHD onset	0	0%
Arterial lactate	0	0%
Hypotension assessment	0	0%
Preload dependence assessment if hypotension	2	3.3%
Time between IHD onset and hypotension	0	0%
Fluid removal at hypotension	0	0%
Heart rate at IHD onset	0	0%
Mean arterial pressure at IHD onset	0	0%
Central venous pressure at IHD onset	2	1.9%
Cardiac index at IHD onset	0	0%
Indexed systemic vascular resistance at IHD onset	2	1.9%
Extravascular lung water index at IHD onset	0	0%
Pulmonary vascular permeability index at IHD onset	7	6.5%
Duration of dialysis session	0	0%
Time between admission and IHD session	0	0%
Total fluid removal during IHD session	0	0%
Dialyzer blood flow rate at IHD onset	0	0%
Dialysate flow at IHD onset	0	0%
Dialysate temperature at IHD onset	0	0%
Dialysate sodium concentration at IHD onset	0	0%

IHD = intermittent hemodialysis; SAPS II = simplified acute physiology score II; SOFA = Sequential Organ Failure Assessment score.
