# Supplementary material for: Prevalence and risk factors of hypotension associated with preload-dependence during intermittent hemodialysis in critically ill patients
Source: Crit Care. 2016 Feb 23;20:44. doi: 10.1186/s13054-016-1227-3 (PMC4765055; doi:10.1186/s13054-016-1227-3)
Supplement: Additional file 3: — Compliance to practice guidelines of intermittent hemodialysis in intensive care units. Description of data: compliance to practice guidelines of intermittent hemodialysis in intensive care units according to reference [2]. Data are number of sessions (percentage of the total number of sessions studied). (PDF 422 kb) [file 13054_2016_1227_MOESM3_ESM.pdf]

File name: Additional file 3

File format: .pdf

Title: Compliance to practice guidelines of intermittent hemodialysis in intensive care units.

Description of data: Compliance to practice guidelines of intermittent hemodialysis in intensive care units according to reference [1]. Data are number of sessions (percentage of the total number of sessions studied).

Dialysis settings	n (%)
Isovolemic connection with 0.9% saline	107 (100%)
Dialysate sodium concentration ≥ 145 mmol/L	75 (70%)
Minimal IHD session duration 4H	92 (86%) *
Dialysate temperature ≤ 37°C	106 (99%)

* IHD sessions of less than 4H00 duration were initially planned for at least a 4H00 duration, but were prematurely interrupted for medical, technical or organizational reasons.

IHD = intermittent hemodialysis.

1. Schortgen F, Soubrier N, Delclaux C, Thuong M, Girou E, Brun-Buisson C, Lemaire F, Brochard L: Hemodynamic tolerance of intermittent hemodialysis in critically ill patients: usefulness of practice guidelines. *Am J Respir Crit Care Med* 2000, **162**(1):197-202.
