# Supplementary material for: Prevalence and risk factors of hypotension associated with preload-dependence during intermittent hemodialysis in critically ill patients
Source: Crit Care. 2016 Feb 23;20:44. doi: 10.1186/s13054-016-1227-3 (PMC4765055; doi:10.1186/s13054-016-1227-3)
Supplement: Additional file 4: — Univariate analysis of risk factors associated with at least one hypotensive episode. Description of data: univariate analysis of risk factors associated with at least one hypotensive episode. (PDF 305 kb) [file 13054_2016_1227_MOESM4_ESM.pdf]

File name: Additional file 4

File format: .pdf

Title: Univariate analysis of risk factors associated with at least one hypotensive episode.

Description of data: Univariate analysis of risk factors associated with at least one hypotensive episode.

Risk factors	Odd ratio	$CI_{95\%}$ of odd ratio	p value
Age (year) *	0.99	[0.94-1.03]	0.62
Male gender	0.70	[0.17-2.31]	0.58
SAPSII *	0.99	[0.95-1.02]	0.46
Reasons for PiCCO® monitoring			0.89
• Septic shock	1		
• Cardiogenic shock	1.08	[0-inf]	
• Other	1.39	[0.32-5.32]	
Time between ICU admission and IHD session (day) *	0.97	[0.92-1.00]	0.05
SOFA score at the day of IHD session *	1.35	[1.12-1.70]	<0.01
Mechanical ventilation at IHD session onset	0.77	[0.27-2.15]	0.62
Inotrope at IHD session onset	0.19	[0.02-1.22]	0.09
Vasopressor at IHD session onset	1.65	[0.60-4.83]	0.33
Vasopressor dose at IHD session onset ($\mu g.kg^{-1}.min^{-1}$) *	18.49	[0.56-4035]	0.13
Heart rate at IHD session onset (min^{-1}) *	1.00	[0.96-1.03]	0.82
MAP at IHD session onset (mm Hg) *	0.90	[0.84-0.96]	<0.001
CVP at IHD session onset (mm Hg) *	0.96	[0.87-1.05]	0.37
CI at IHD session onset ($L.min^{-1}.m^{-2}$) *	0.91	[0.43-1.68]	0.76
ISVR at IHD session onset ($dyne.s.cm^{-5}$) †	0.78	[0.47-1.27]	0.31
EVLWI at IHD session onset ($ml.kg^{-1}$ PBW) *	0.88	[0.71-1.05]	0.16
PVPI at IHD session onset *	1.11	[0.37-3.46]	0.85
Preload dependence at IHD session onset	3.25	[0.34-80]	0.32
Arterial lactates ($mmol.L^{-1}$) *	1.19	[0.64-2.29]	0.57
Elevated lactates above upper laboratory limit (2.2	2.41	[0.58-11.84]	0.23

mmol.L^{-1})			
Duration of IHD sessions (min) †	1.49	[0.91-2.70]	0.12
Total fluid removal during IHD session (mL) †	0.83	[0.50-1.37]	0.45
Fluid removal per hour during IHD session (mL.H^{-1}) †	0.78	[0.46-1.29]	0.32
Dialyzer blood flow rate at IHD session onset (L.min^{-1}) †	1.31	[0.83-2.19]	0.25
Dialysate flow at IHD session onset (mL.min^{-1}) †	1.41	[0.87-2.35]	0.16
Dialysate temperature at IHD session onset (°C) †	0.95	[0.58-1.53]	0.83
Dialysate sodium concentration at IHD session onset (mmol.L^{-1}) †	1.58	[0.96-2.88]	0.07

* per one unit increase, † per one standard deviation increase~~*~~.

CI = cardiac index; $CI_{95\%}$ = 95% confidence interval; CVP = central venous pressure; MAP = mean arterial pressure; EVLWI = extravascular lung water; IHD = intermittent hemodialysis; inf = infinity; ISVR = indexed systemic vascular resistance; PBW = predicted body weight; PVPI = pulmonary vascular permeability index; SAPSII = simplified acute physiology score II; SOFA = Sequential Organ Failure Assessment score.
