# Supplementary material for: Prevalence and risk factors of hypotension associated with preload-dependence during intermittent hemodialysis in critically ill patients
Source: Crit Care. 2016 Feb 23;20:44. doi: 10.1186/s13054-016-1227-3 (PMC4765055; doi:10.1186/s13054-016-1227-3)
Supplement: Additional file 5: — Hypotension rates during intermittent hemodialysis in intensive care units in published studies. Description of data: hypotension rates during intermittent hemodialysis in intensive care units in published studies. (PDF 113 kb) [file 13054_2016_1227_MOESM5_ESM.pdf]

File name: Additional file 5

File format: .pdf

Title: Hypotension rates during intermittent hemodialysis in intensive care units in published studies.

Description of data: Hypotension rates during intermittent hemodialysis in intensive care units in published studies.

Study	Year of publication	Hypotension rate	SAPS II	SOFA	Vasopressor requirement	Mechanical ventilation	Criteria defining hypotension
Schortgen (1) *	2000	56%	59	NR	34%	67%	SAP drop from baseline value > 10%
Tonelli (2)	2002	18%	NR	NR	30%	85%	MAP < 70 mm Hg for at least 5 minutes
Vinsonneau (3)	2006	39%	64	NR	86%	95%	SAP < 80 mm Hg or SAP drop from baseline > 50 mm Hg
Du Cheyron (4)	2013	17%	57	8	22%	34%	SAP < 90 mm Hg justifying therapeutic interventions
Present study	-	57%	53	8	50%	36%	MAP < 65 mm Hg

* intervention arm with dedicated IHD guidelines to improve hemodialysis tolerance.

ICU = intensive care units; IHD = intermittent hemodialysis; MAP = mean arterial pressure; NR = not reported; SAP = systolic arterial pressure; SAPS II = simplified acute physiology score; SOFA = Sequential Organ Failure Assessment score

1. Schortgen F, Soubrier N, Delclaux C, et al: Hemodynamic tolerance of intermittent hemodialysis in critically ill patients: usefulness of practice guidelines. *Am J Respir Crit Care Med* 2000;162:197-202

2. Tonelli M, Astephen P, Andreou P, et al: Blood volume monitoring in intermittent hemodialysis for acute renal failure. *Kidney Int* 2002;62:1075-1080

3. Vinsonneau C, Camus C, Combes A, et al: Continuous venovenous haemodiafiltration versus intermittent haemodialysis for acute renal failure in patients with multiple-organ dysfunction syndrome: a multicentre randomised trial. *The Lancet* 2006;368:379-385

4. du Cheyron D, Terzi N, Seguin A, et al: Use of online blood volume and blood temperature monitoring during haemodialysis in critically ill patients with acute kidney injury: a single-centre randomized controlled trial. *Nephrol Dial Transplant* 2013;28:430-437
